# Mechanical Strain Downregulates C/EBPβ in MSC and Decreases Endoplasmic Reticulum Stress

**DOI:** 10.1371/journal.pone.0051613

**Published:** 2012-12-12

**Authors:** Maya Styner, Mark B. Meyer, Kornelia Galior, Natasha Case, Zhihui Xie, Buer Sen, William R. Thompson, John Wesley Pike, Janet Rubin

**Affiliations:** 1 Department of Medicine, University of North Carolina, Chapel Hill, North Carolina, United States of America; 2 Department of Biochemistry, University of Wisconsin-Madison, Madison, Wisconsin, United States of America; Georgia Health Sciences University, United States of America

## Abstract

Exercise prevents marrow mesenchymal stem cell (MSC) adipogenesis, reversing trends that accompany aging and osteoporosis. Mechanical input, the in-vitro analogue to exercise, limits PPARγ expression and adipogenesis in MSC. We considered whether C/EBPβ might be mechanoresponsive as it is upstream to PPARγ, and also is known to upregulate endoplasmic reticulum (ER) stress. MSC (C3H10T1/2 pluripotent cells as well as mouse marrow-derived MSC) were cultured in adipogenic media and a daily mechanical strain regimen was applied. We demonstrate herein that mechanical strain represses C/EBPβ mRNA (0.6-fold ±0.07, p<0.05) and protein (0.4-fold ±0.1, p<0.01) in MSC. SiRNA silencing of β-catenin prevented mechanical repression of C/EBPβ. C/EBPβ overexpression did not override strain’s inhibition of adipogenesis, which suggests that mechanical control of C/EBPβ is not the primary site at which adipogenesis is regulated. Mechanical inhibition of C/EBPβ, however, might be critical for further processes that regulate MSC health. Indeed, overexpression of C/EBPβ in MSC induced ER stress evidenced by a dose-dependent increase in the pro-apoptotic CHOP (protein 4-fold ±0.5, p<0.05) and a threshold reduction in the chaperone BiP (protein 0.6-fold ±0.1, p = 0.2; mRNA 0.3-fold ±0.1, p<0.01). ChIP-seq demonstrated a significant association between C/EBPβ and both *CHOP* and *BiP* genes. The strain regimen, in addition to decreasing C/EBPβ mRNA (0.5-fold ±0.09, p<0.05), expanded ER capacity as measured by an increase in BiP mRNA (2-fold ±0.2, p<0.05) and protein. Finally, ER stress induced by tunicamycin was ameliorated by mechanical strain as demonstrated by decreased C/EBPβ, increased BiP and decreased CHOP protein expression. Thus, C/EBPβ is a mechanically responsive transcription factor and its repression should counter increases in marrow fat as well as improve skeletal resistance to ER stress.

## Introduction

Exercise increases skeletal strength through mechanical effects that improve bone mineral content and architecture [1]–[3]. The positive effect of exercise on the skeleton depends, at least partially, on the ability of mechanical input to regulate output of osteoblasts from progenitor mesenchymal stem cells (MSC). Decreased adipocytes and increased pre-osteoblasts have been demonstrated in the marrow of running rats [4] and climbing mice [5], indicating that MSCs are targeted by mechanical input. MSC adipogenesis, recapitulated *in-vitro*, is highly sensitive to mechanical loading [6]. We have shown that mechanical input applied to MSC slows adipogenesis in a process marked by downregulation of PPARγ as well as activation of β-catenin [6]–[8].

Several studies demonstrate that negative regulators of adipogenesis exert their effects via a critical transcription factor upstream of PPARγ, C/EBPβ [9]–[13]. Nutrients, hormones, and genetic factors induce C/EBPβ, which upregulates both PPARγ and its dimeric transcription partner, C/EBPα [14], [15]_ENREF_23. As mechanical input leads to reduced expression of PPARγ [6], we hypothesized that mechanical signals might regulate C/EBPβ.

Besides its role in PPARγ induction, C/EBPβ plays a key role in the induction of endoplasmic reticulum (ER) stress [16], [17]. A functioning ER has been shown to be important to the health of virtually all cells and tissues [18]. Exercise induced mechanical factors have been shown to ameliorate ER stress in the brains of running mice [19], in loaded mouse ulnae [20], and in osteoblasts [21]. The ER stress response of stem cells has not been well studied, but resistance to ER stress has been associated with maintenance of pluripotency [22] and differentiation [23]. Notably, ER stress pathways, and in particular the PERK arm, have been implicated in skeletal health [24], [25]: PERK knockout mice have skeletal defects and a PERK functional haplotype is associated with lower bone mineral density in humans [24], [25]. ER stress induces transcription of multiple responders, such as BiP, a chaperones which can ameliorate the response to stress, and CHOP, which alters ER protein translation and is pro-apoptotic [26]. We will demonstrate herein that mechanical input, in addition to repressing C/EBPβ, which is required for adipogenesis, also decreases expression the apoptotic CHOP while increasing the expression of the chaperone BiP in MSC, suggesting novel mechanisms for the beneficial effects of exercise on skeletal health.

## Methods

### Ethics Statement

All animal work was conducted according to relevant national and international guidelines. Wild Type C57/BL6 mice were used for the isolation of marrow-derived MSC (mdMSC). This animal work was approved by the University of North Carolina Animal Care and Use Committee (IACUC). The mice were sacrificed via CO2 and University of North Carolina-approved physical disruption method. Appropriate steps were taken to ameliorate suffering in accordance with our institutional IACUC.

### Reagents

Culture medium, antibiotics, trypsin-EDTA, reverse transcriptase, siRNA, MgCl_2_, 10×PCR buffer, and Taq polymerase were obtained from Invitrogen (Carlsbad, CA). Fetal bovine serum was from Atlanta Biologicals (Atlanta, GA). Insulin, indomethacin, dexamethasone, and SB415286 were from Sigma-Aldrich (St. Louis, MO). LipoD293 and PepMute were from SignaGen (Ijamsville, MD).

### Cell Culture

C3H10T1/2 pluripotent stem cells or mouse marrow-derived MSC (mdMSC) were maintained in growth medium (α-MEM along with 10% FBS and 100 µg/ml penicillin/streptomycin). Cells were plated in six-well plates at a density of 1×10^5^ except as otherwise noted one day prior to initiation of experiments. Adipogenic medium including 0. 1 µM dexamethasone, 50 µM indomethacin, and 5 µg/ml insulin was added on day zero. Key experiments in the C3H10T1/2 cells were replicated in a marrow derived MSC line generated from C57/BL6 wild-type mice using the procedure of [27], [28]. These cells readily undergo differentiation into osteogenic, adipogenic or alternative lineages using standard differentiation media [28]. We have termed these cells “marrow derived MSC” (mdMSC) in the text. Early adipogenesis is measurable in mdMSC a day earlier than in the C3HT101/2 cells under the same conditions and thus the timing of adipogenesis experiments was adjusted to reflect this as noted in figure legends.

### Mechanical Input

Daily strain regimens (2% magnitude, 0.17 Hz, 3600 cycles/day or 300 cycles twice/day) previously shown to repress MSC adipogenesis [6], [29] were applied using the Flexcell FX-4000 system (Flexcell International, Hillsborough, NC). For adipogenesis experiments, strain was applied each day in culture as indicated on figure legends.

### RNA Interference

MSC were transfected at 50% confluence with siRNA (20 nM; Invitrogen) in DMEM-high glucose media for 6 hours using PepMute siRNA reagent per the manufacturer’s protocol. Protein isolation was performed 72 hours after transfection. C/EBPβ siRNA sequence was as follows: 5′-UUG-GCC-ACU-UCC-AUG-GGU-CUA-AAGG-3′.

### Protein Analysis

Whole cell lysates were prepared and western blotting performed as previously described in [30]. Antibodies for immunoblotting include: active β-catenin (clone 8E7, Upstate, Temecula, CA), total β-catenin (BD, Bedford, MA), adiponectin, β-tubulin, BiP, and CHOP (Santa Cruz Biotechnology, CA), aP2 (ProSci, Inc., Poway, CA), C/EBPβ, phospho-C/EBPβ (Thr235), phospho-GSK3β (serine-9), PPARγ (Cell Signaling, Danvers, MA), and GSK3β (Chemicon, Billerica, MA). Densitometry was determined using NIH ImageJ, 1.37v.

### Real Time PCR

Total RNA was isolated and one microgram was reverse transcribed and analyzed via real time PCR as previously described [30]. Ten microliters of cDNA from each experimental condition were pooled and diluted 1∶10, 1∶100, 1∶1,000 and 1∶10,000 to generate a 5-point standard curve. A non-template control was added to each PCR reaction. PPARγ2, adiponectin, and 18S primers were as in [6], [31] and C/EBPβ primers were as in [32]. Standards and samples were run in duplicate. PCR products were normalized to 18S amplicons.

### Plasmids and Transfection

The pcDNA3.1 (−) mouse C/EBPβ plasmid was obtained from Addgene (plasmid 12557, deposited by Peter Johnson). 2.5×10^5^ cells were seeded in 6-well plates in triplicate. On day zero, cells were transfected with pcDNA3.1 (−)C/EBPβ at indicated concentrations versus pcDNA3.1 expression vector using the LipoD293 reagent per manufacturer protocol at 3 µl per 1 µg of DNA. Salmon sperm DNA was added to equalize microgram of DNA transfected per experimental condition (Figure 3B–C). Transfection reagent was applied at the same concentration for each experimental condition.

### Chromatin Immunoprecipitation

MC3T3 cells as well as mdMSC were treated for 3 hours with vehicle or 50 mM LiCl followed by chromatin immunoprecipitation performed as described previously [33]–[36]. Briefly, Isolated DNA [or DNA acquired before precipitation (input)] was subjected to further preparation for ChIP-seq analysis and results were additionally confirmed by quantitative real-time PCR (qPCR). Antibodies to β-catenin (H-102, sc-7199; C-18, sc-1496; E-5, sc-7963) were purchased from Santa Cruz Biotechnology, Inc. (Santa Cruz, CA). TCF-4 (clone 6H5–3) antibody was purchased from Millipore Corp. (Billerica, MA). ChIP-seq was performed as previously described in [37].

### Statistical Analysis

Results are expressed as mean±SEM. Statistical significance was evaluated by t-test, one-way ANOVA with a Tukey’s post hoc test, or two-way ANOVA with a Bonferroni posttest (GraphPad Prism 5.04). All experiments were replicated at least twice to assure reproducibility. Statistical significance is indicated on graphs and figure legends.

## Results

### C/EBPβ is a Mechanical Target in MSC

**Figure 1:**
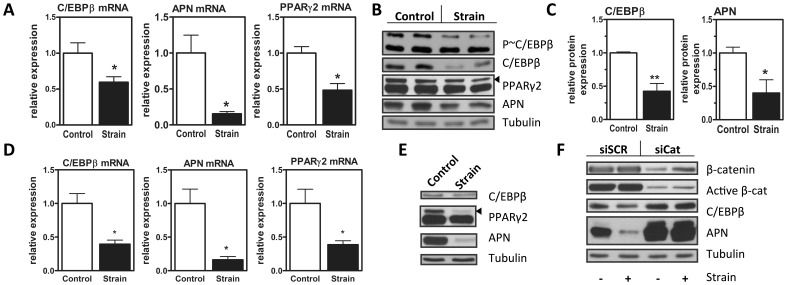
C/EBPβ is a mechanical target in MSC. A, mRNA of C3H10T1/2 cells in adipogenic media subjected to strain, analyzed via real time PCR for C/EBPβ, adiponectin (APN), and PPARγ2, n = 3, *p<0.05. B, Immunoblot of day 3 C3H10T1/2 cells in adipogenic media subjected to strain. C, Densitometry from (B) n = 4, * p<0.05, ** p<0.01. D, mRNA of mdMSC in adipogenic media treated with strain, analyzed via real time PCR for C/EBPβ, adiponectin (APN), and PPARγ2, n = 3,*p<0.05. E, Representative immunoblot of day 2 mdMSC cells in adipogenic media subjected to strain, n = 3. F, C3H10T1/2 cells transiently transfected with siRNA to β-catenin. 24 hr later, induction of adipogenesis was initiated with adipogenic media. Strain was applied and immunoblot performed for β-catenin, active β-catenin, as well as C/EBPβ, and adiponectin (APN).

We have previously shown that mechanical input inhibits the expression of PPARγ protein in MSC, suggesting that there should be a previously unidentified mechanically sensitive target proximal to PPARγ [6]. Since C/EBPβ is activated early in adipogenesis and induces expression of PPARγ, we queried whether mechanical strain might regulate expression of C/EBPβ. Application of mechanical strain to cells cultured in adipogenic medium significantly limited the rise in C/EBPβ mRNA expression which occurred in early adipogenesis, compared to unloaded control cultures (p<0.05, see **figure 1A**). Along with the significant repression of C/EBPβ mRNA we measured decreases in expression of fat markers adiponectin (p<0.05) and PPARγ (p<0.05). Mechanical inhibition of C/EBPβ mRNA predicted reductions of C/EBPβ protein as well as PPARγ2 and adiponectin (**figure 1B**). Densitometry confirmed that mechanical repression of C/EBPβ and adiponectin proteins were significant (**figure 1C**, p<0.01 and p<0.05, respectively). As C/EBPβ is activated by phosphorylation at a consensus ERK/GSK3β site, we next explored whether mechanical strain alters C/EBPβ activation. The reduction in phospho-C/EBPβ, the active form of this transcription factor, correlated with reduced expression of total C/EBPβ and was not observed to be greater than the effect on total C/EBPβ expression (**figure 1B**), suggesting that mechanical repression of C/EBPβ is at a transcriptional level.

Key findings in C3H10T1/2 cells were reproduced in mouse marrow-derived MSC (mdMSC). Similar to the mechanical effect in C3H10T1/2 cells, C/EBPβ mRNA and protein were downregulated by mechanical input in mdMSC at day 3 of adipogenic differentiation (**figure 1D** **,** **2E**). As well, PPARγ and adiponectin mRNA and protein were decreased in the presence of mechanical stimulation.

**Figure 2:**
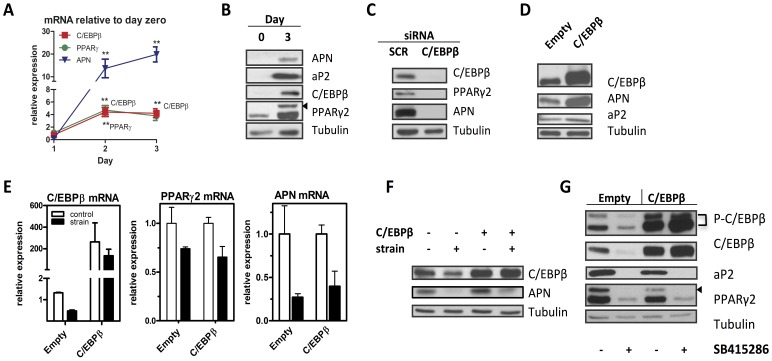
C/EBPβ overexpression does not rescue adipogenesis from mechanical inhibition. A, C3H10T1/2 cells cultured in adipogenic media and analyzed on indicated days by real time PCR for C/EBPβ, PPARγ, and adiponectin (APN) n = 3, **p<0.01 relative to day zero B, C3H10T1/2 cells cultured in adipogenic media and blotted for C/EBPβ, markers of adipogenesis. C, Immunoblot of C3H10T1/2 cells transfected with siRNA to C/EBPβ. 24 hours after transfection, adipogenic medium was added for 72 hours. D, C3H10T1/2 cells were transfected with 1 µg of overexpression vector for C/EBPβ or control empty vector. 24 later adipogenic media was added and cells were analyzed at 48 hours by immunoblot for C/EBPβ, adiponectin (APN) or aP2. E, C3H10T1/2 cells overexpressing C/EBPβ as in A were subjected to strain for 3 days and analyzed by real-time PCR for C/EBPβ, PPARγ2 and adiponectin mRNA expression. F, experiment as described in (E) analyzed by immunoblot. G, C3H10T1/2 cells were transiently transected with a C/EBPβ overexpression vector. 24 hours later adipogenic media +/−SB41528 was added and maintained for 3 days prior to protein analysis for markers of adipogenesis and C/EBPβ.

β-catenin contributes significantly to strain repression of adipogenesis [7]. We asked if β-catenin participated in mechanical regulation of C/EBPβ. SiRNA targeting β-catenin or a control siRNA sequence was transfected into cultures prior to induction of adipogenesis. In cultures treated with control siRNA, mechanical strain had expected effects to repress adipogenesis, shown here by reduced expression of adiponectin (**figure** **1F**, **left lanes**). As in **figure 1A, 1B**, mechanical strain repressed C/EBPβ protein expression (**figure 1F**). Knockdown of β-catenin (**figure 1F** **, right lanes**) accelerated adipogenesis as shown by increased expression of adiponectin. β-catenin knockdown interfered not only with mechanical repression of adiponectin, but also prevented the mechanical inhibition of C/EBPβ. Thus, C/EBPβ is mechanically sensitive and predicts that multiple C/EBPβ targets, such as PPARγ expression and the ER stress response should be susceptible to mechanical input.

### C/EBPβ Overexpression does not Rescue Adipogenesis from Mechanical Inhibition

Having established C/EBPβ as a mechanical target, we sought to determine if C/EBPβ was necessary for mechanical repression of adipogenesis. Adipogenic differentiation of MSC was marked by an early expression peak of C/EBPβ mRNA at day 2 (**figure 2A**) and protein at day 3 (**figure 2B**). The rise in C/EBPβ was noted concomitantly with the expression of PPARγ as well as PPARγ target genes adiponectin and aP2. Consistent with prior studies in 3T3-L1 pre-adipocytes [38], knockdown of C/EBPβ with siRNA prevented adipogenesis of multipotential MSC (**figure 2C**). To investigate whether mechanical repression of C/EBPβ was critical for mechanical inhibition of adipogenesis, we studied adipogenesis during C/EBPβ overexpression (**figure 2D** **)**. As C/EBPβ does not induce adipogenesis alone, adipogenic medium was added to cultures transfected with a C/EBPβ expression or empty vector. C/EBPβ overexpression enhanced adipogenesis (**figure 2D**).

MSC were treated with a daily mechanical regimen for 3 days during adipogenic culture. The mechanical regimen effectively inhibited adipogenesis, as measured by decreases in adiponectin and PPARγ2 mRNA as well as adiponectin protein, despite overexpression of C/EBP β (**figure 2E, F**). We additionally asked if pharmacological β-catenin activation could overcome C/EBPβ-mediated adipogenesis. SB415286, a GSK3β inhibitor, was added to cultures undergoing adipogenesis, causing a reduction in in adipogenic differentiation as measured by PPARγ and aP2 protein (**figure 2G** **)**. The effect of SB41528 to repress adipogenesis, similar to that of mechanical strain, was maintained in the presence of C/EBPβ overexpression **(** **figure 2G** **, right lanes)**. As such, mechanical repression of adipogenesis was not rescued by C/EBPβ. Despite this result, the combination of our data showing that C/EBPβ is not, alone, enough to stimulate adipogenesis of MSC, and our confirmation that adipogenesis requires C/EBPβ expression, supports that that repression of C/EBPβ must contribute to the anti-adipogenic effects of mechanical stimulation. In addition to the importance of our findings for adipogenic differentiation, we explored other potentially salutary downstream effects of C/EBPβ downregulation.

### C/EBPβ is Involved in ER Stress in MSC

**Figure 3:**
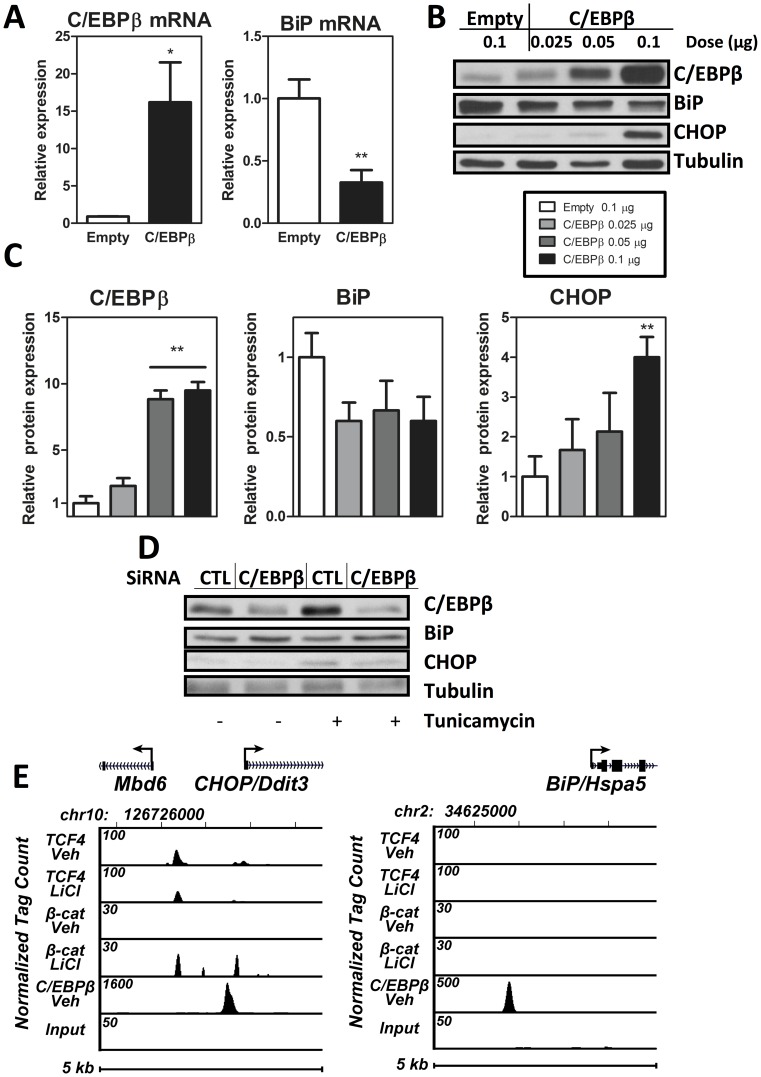
C/EBPβ is involved in ER stress in MSC. A, C3H10T1/2 cells were transfected with 1 µg of C/EBPβ versus empty vector. 48 hours after transfection, RNA analysis was performed via real time PCR (n = 5). B, C3H10T1/2 cells transiently transfected with C/EBPβ versus empty vector at indicated doses and 48 hours after transfection, protein analysis was performed. A representative blot is shown, n = 3. C, Densitometry for (B). D, Immunoblot of C3H10T1/2 cells transfected with siRNA to C/EBPβ or control siRNA shown at 72 hours after transfection. Tunicamycin at 1 µg/ml was added for the final 6 hours. E, ChIP-seq analysis of C/EBPβ, β-catenin, TCF4 binding at the mouse *CHOP* and *BiP* gene loci. MC3T3 cells were treated for 3 hours with vehicle or 50 mM LiCl prior to ChIP-seq assay. The genomic interval on the indicated mouse chromosome and the location of the *CHOP* or *BiP* transcription unit including the direction of transcription (arrow) is shown at the top. Tracks indicate tag densities (normalized to 10^7^ reads) for vehicle or LiCl treated β-catenin or TCF4 binding. Note the scale for peak height is different for each track to highlight peak activities.

As C/EBPβ has been shown to trigger endoplasmic reticulum stress in pancreatic β-cells and fibroblasts [16], [17], we considered whether C/EBPβ might play a similar role in the MSC unfolded protein response. We first examined the effect of C/EBPβ overexpression on components of the ER stress response. C/EBPβ overexpression downregulated the ER chaperone BiP mRNA (**figure 3A**; p<0.01). Concomitant with the reduction in BiP, C/EBPβ overexpression upregulated the pro-apoptotic CHOP protein in a C/EBPβ-dose-dependent manner (**figure 3B**; p<0.01) as confirmed by densitometry (**figure 3C**). Moreover, ablation of C/EBPβ by siRNA decreases expression of CHOP and increases expression of BiP (**figure 3D**). As BiP confers improved capacity to resist ER stress [39], a reduction of BiP in the presence of C/EBPβ overexpression is consistent with a reduction in the cells ability to withstand ER stress. C/EBPβ upregulation of the pro-apoptotic CHOP-as well as the diminution of CHOP with siRNA to C/EBPβ- additionally implicates C/EBPβ in exacerbation of ER stress.

We then asked whether C/EBPβ might directly regulate expression of these two components of the ER stress response- BiP and CHOP. Indeed, ChIP-seq analysis demonstrated that C/EBPβ is significantly associated with both *BiP* and *CHOP* genes (**figure 3E**). As mechanical regulation of C/EBPβ required β-catenin, we queried if β-catenin might associate with either *BiP* or *CHOP* genes in a ChIP-seq analysis. Neither β-catenin nor its associated transcription factor TCF4 showed significant association with the upstream promoter of *BiP*. There was an association of β-catenin and TCF4 with *CHOP*; however this association was significantly lower than that of C/EBPβ with the *CHOP* gene. This suggests that although β-catenin is important for repression of adipogenesis, C/EBPβ regulatory role in ER stress is unlikely to involve direct effects of β-catenin on these responders. Thus, C/EBPβ might affect ER stress by direct association of the transcription factor with the *CHOP* and *BiP* genes, regulating their expression levels in MSC.

### Mechanical Strain Increases ER Capacity in MSC

**Figure 4:**
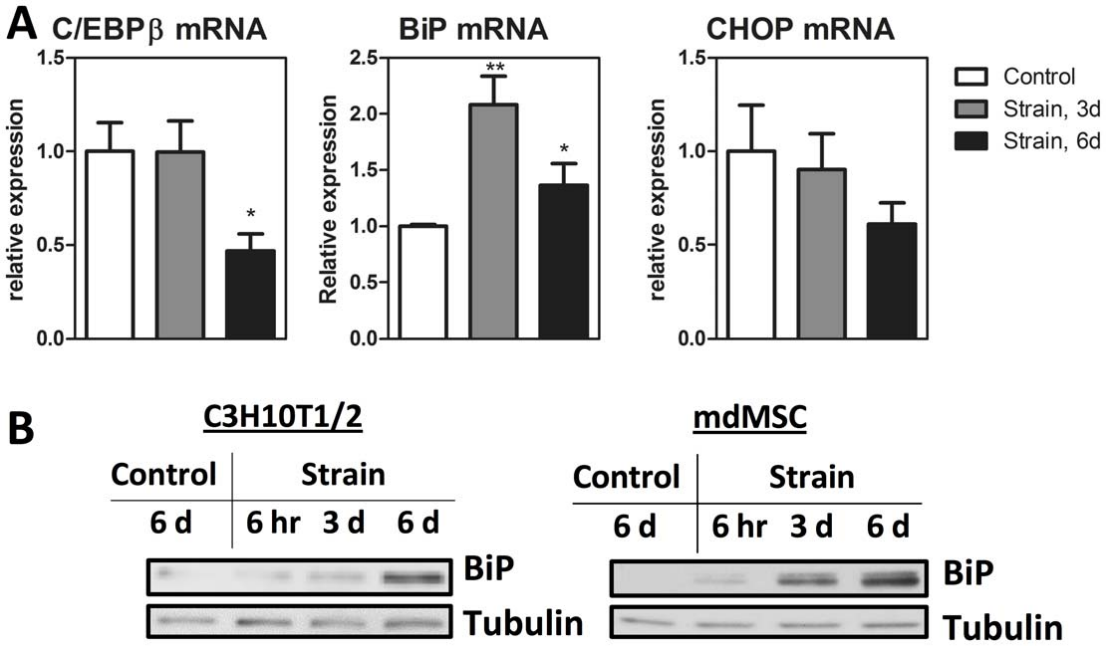
Mechanical strain increases ER capacity in MSC. A**,** MSC seeded on the same day were treated with mechanical strain for indicated time prior to mRNA (n = 3 * P<0.05, ** P<0.01) (A) or protein analysis (B) performed with C3H10T1/2 cells and mdMSC, as indicated. Control cultures were seeded on the same day as strained cultures and housed in similar conditions for 6 days prior to analysis.

The C/EBPβ effect to decrease BiP and increase CHOP suggests that it impairs the MSC’s ability to resist ER stress. Indeed, in pancreatic β-cells, ablation of C/EBPβ has been demonstrated to improve ER capacity [16]. We were thus interested ascertain if mechanical repression of C/EBPβ would similarly improve ER capacity in MSC. We found that a daily mechanical strain regimen increased BiP mRNA compared to non-strained controls at both 3 and 6 days of a daily mechanical regimen (**figure 4A**; p<0.01 and p<0.05). Consistent with work demonstrating that endurance exercise ameliorates ER stress in muscle [40], increases in BiP protein were found in both C3H10T1/2 and mdMSC after an *in-vitro* endurance regimen with increased benefit at 6 days as compared to either 3 days or 6 hours (**figure 4B**). CHOP mRNA in these cultures showed a trend toward reduction at 6 days, although CHOP protein was not measurable in unstressed cultures. Thus, mechanical strain in the setting of adipogenesis (figure 1) downregulates C/EBPβ after 3 days as compared to 6 days required to downregulate C/EBPβ in non-adipogenic conditions (figure 4A). This difference is likely due to early adipogenic C/EBPβ induction (see figure 2A) that allows strain effects to be demonstrable at an earlier time point. Consistent with an expansion of the ER as marked by increased BiP, CHOP mRNA expression was decreased in cultures which were treated daily with mechanical strain.

### Mechanical Strain Ameliorates Tunicamycin-induced ER Stress in MSC

**Figure 5:**
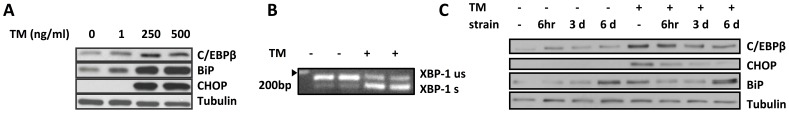
Mechanical strain ameliorates tunicamycin-induced ER stress in MSC. A, mdMSC were cultured in the presence of tunicamycin (TM) at indicated doses for 6 hours and analyzed via immunoblot for C/EBPβ, BiP and CHOP. B, as in (A) mdMSC cultured in the presence of tunicamycin 1 µg/ml for 6 hours and analyzed via conventional PCR for XBP1. C, C3H10T1/2 cells were seeded on the same day and treated with mechanical strain for the indicated number of days. Tunicamycin (TM) was added for the final 6 hours prior to protein analysis (representative blot, n = 3).

We next tested whether mechanical induction of BiP could afford protection from ER stress. Endoplasmic reticulum stress was induced with tunicamycin, which blocks synthesis of N-linked glycoproteins, leading to an accumulation of protein in the ER lumen. Tunicamycin dose-dependently increased the expression of C/EBPβ, as well as that of CHOP and BiP in MSC (**figure 5A**). Additionally tunicamycin induced alternative splicing of XBP1 in MSC (**figure 5B**) consistent with activation of a second arm of the unfolded protein response [26]. Mechanical strain applied for 6 hours, 3 days or 6 days effectively ameliorated tunicamycin-induced ER stress as evidenced by decreased C/EBPβ protein as well as increased BiP and decreased CHOP (**figure 5C**). Increasing the days of mechanical treatment prior to tunicamycin treatment increased this beneficial response. C/EBPβ transmits deleterious effects of ER stress [16] and enables adipogenesis. In Figure 5C, the small rise in C/EBP β measured in strained cells conflicts with the significant repression of C/EBPβ expression shown in figure 2 and 4. It is likely that mechanical effects on expression of this gene are context dependent: we have reproducibly measured, here, the ability of mechanical strain to repress stimulated rises in C/EBPβ. Thus, repetitive mechanical input, akin to endurance exercise, improved MSC ability to resist a significant ER stressor.

## Discussion

Exercise is critical to musculoskeletal health. Mechanical stimulation biases the bone marrow MSC away from the fat lineage, permitting stem cell entry into other higher order tissues [6], [7], [28], [31]. When daily mechanical loads are decreased – as in paralysis or in microgravity of spaceflight - osteopenia rapidly ensues [41], [42]. We have now shown that mechanical input limits expression of the C/EBPβ, a transcription factor central for processes associated with reduced skeletal health: adipogenesis and endoplasmic reticulum stress [16], [24], [25], [38].

C/EBPβ regulates adipogenesis at multiple levels. C/EBPβ shares significant target gene-binding homology with C/EBPα as well as PPARγ, and the loss of any one of these factors interferes with the adipogenic program [43]. Recent work suggests that a majority of adipocyte genes are not regulated by PPARγ alone but require simultaneous actions of C/EBPα and C/EBPβ rather than individual actions in discrete sequential steps [43]. As such, mechanical control of C/EBPβ can be expected to contribute to repression of early steps of adipogenesis. The failure of C/EBPβ overexpression to prevent mechanical inhibition of adipogenesis suggests that its contribution is less than other adipogenic factors that are subject to mechanical control. For example, mechanically activated β-catenin downregulates PPARγ activity [44], and β-catenin is activated by mechanical stimuli [45]. Another potential mechanism is the known effect of mechanical input to stimulate MAPK, which can phosphorylate and repress PPARγ-directed transcription [46]. As such mechanical effects, including those due to preservation and activation of β-catenin, act at multiple sites. This also suggests that the mechanical regulation of C/EBPβ may be important for non-adipogenic functions of this widely expressed transcription factor.

That C/EBPβ is subject to mechanical control further widens the net of cellular events subject to biophysical control. In addition to its role in adipogenesis, this transcription enhancer plays a role in immune responses [47], [48], cancer [49], hepatocyte differentiation [50], and has notably been shown to upregulate ER stress [16]. BiP buffers the cell from ER stress by providing docking sites for ATF6, IRE1 and PERK [26]. When these three ER transducers are released, downstream effectors traffic from the ER to the nucleus, triggering apoptosis via a rise in CHOP out of proportion to BiP [51]–[53]. Increased expression of C/EBPβ can accelerate negative aspects of the unfolded protein response [16], [17] activating CHOP and pro-apoptotic caspase-3. In MSCs, we have now shown that overexpression of C/EBPβ increases CHOP expression, and decreases BiP, thus decreasing cellular capacity for handling of ER stress.

Besides increasing transcription of CHOP, C/EBPβ serves as the principal dimerization partner for the pro-apoptotic CHOP [17]. As such, C/EBPβ overexpression in pancreatic β cells was shown to increase these pro-apoptotic proteins while decreasing the beneficial chaperone BiP [16]. Conversely, ablation of C/EBPβ resulted in an increased ER capacity, reflected by increased BiP. Accordingly, C/EBPβ deficient MSC show resistance to tunicamycin-induced apoptosis [16]. In line with these studies, we found that mechanical repression of C/EBPβ correlates with reduced ER stress in MSC, suggesting that mechanical input may have salutary effects that are transmitted through a C/EPBβ control point.

Accumulating evidence suggests that exercise serves to lower ER stress by preserving and enhancing ER capacity [19], [20]. For example, exercise downregulates C/EBPβ in cardiomyocytes and ablation of C/EBPβ in trained mice improves resistance to a heart-specific pathological stress [54]. Our data is the first to suggest that for MSCs, mechanical inhibition of C/EBPβ promotes MSC cell health through increasing resistance to stress. Furthermore, we have identified that C/EBPβ is an important mechanical target in MSC, and is likely to be in other tissue where C/EBPβ controls cell differentiation and function. Thus, C/EBPβ is a mechanically responsive transcription factor and its biophysical control advances our understanding of how exercise is beneficial to MSC/skeletal health.
